# Minimally Invasive Vacuum-Aided Extraction Technique for the Lipid Analysis of Historic Parchment

**DOI:** 10.1021/acs.analchem.4c01395

**Published:** 2024-08-16

**Authors:** Samuel P. Johns, Charlie A. Maule, Lora Angelova, Marc Vermeulen, Chris Day, Marta Muñoz-Alegre, Matthew J. Collins, Mélanie Roffet-Salque

**Affiliations:** † Organic Geochemistry Unit, School of Chemistry, University of Bristol, Cantock’s Close, Bristol BS8 1TS, U.K.; ‡ Collection Care Department, The National Archives, Bessant Drive, Richmond TW9 4DU, London, U.K.; § Collection Expertise and Engagement Department, The National Archives, Bessant Drive, Richmond TW9 4DU, London, U.K.; ∥ McDonald Institute for Archaeological Research, University of Cambridge, Downing Street, Cambridge CB2 3ER, U.K.; ⊥ The Globe Institute, University of Copenhagen, Oster Voldgade 5-7, 1353 Copenhagen, Denmark

## Abstract

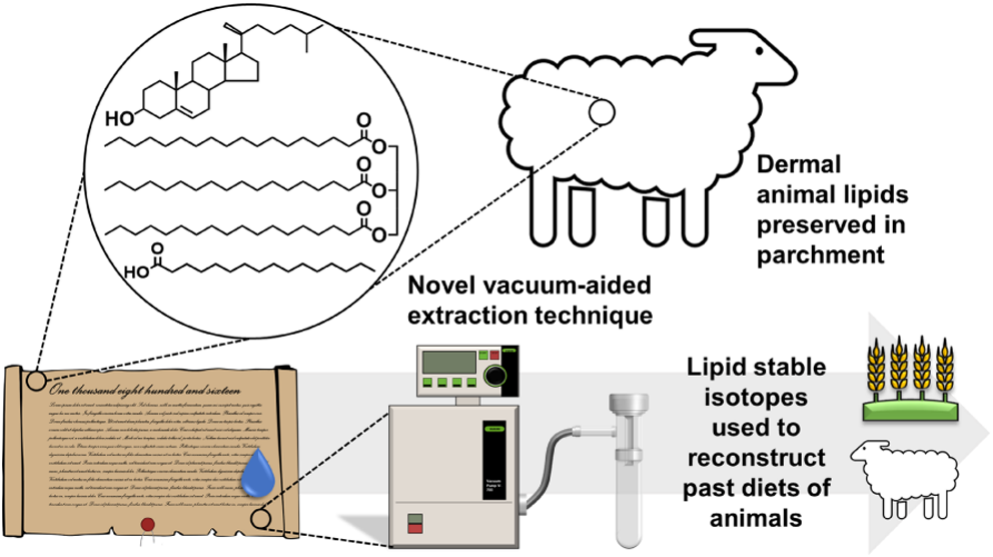

Parchment is an ancient writing support formed from dehaired animal skins. Its manufacture comprises a series of liming and scraping steps before being stretched and dried under tension. Historical parchment represents a valuable source of cultural heritage which, until now, has limited investigations to noninvasive analyses to infer ink composition, degradation, or physical changes over time. We highlight the prospect of the molecular and isotope compositions of animal lipids from parchment as an untapped record of its production and the animal’s diet and environment. We report a minimally invasive, total lipid extraction aided by a vacuum for historical parchments. The quantitative and qualitative compositions of lipid extracts obtained using this method are compared with those obtained using invasive sampling for nine sacrificial membranes dated 1765–1825 CE. This extraction method is then applied to membranes from the Chancery Parliament Rolls (1814–1820 CE) held by The National Archives, UK to obtain lipids and derive taxonomic and dietary information using their stable carbon isotope compositions. This novel vacuum-aided extraction allows, for the first time, animal lipids to be obtained from parchment minimally invasively, paving the way for dietary and paleoclimate studies using this well-dated and common material.

Parchment is a biological material made from processed animal skins, predominantly goat, sheep, and calf. Its manufacture has been influenced by societal and geographical nuances;^1−4^ but common to all methods is the depilation of untanned skins by repeated exposure to an alkaline liming solution, mechanical fleshing, and stretching over a frame to dry under tension.^5,6^ The resulting product is a robust membrane that has enabled the documentation of litigation, art, and societal information.^2^ Historic parchment, therefore, represents a highly valuable record of cultural heritage and, unlike many archeological materials, is ubiquitous, commonly well-curated, and often well-dated as it is a statement of record. The legal and cultural importance of parchment has meant that manuscript studies are often constrained to noninvasive techniques that seek to characterize organic and inorganic components of ink/pigment pallets,^7−13^ biodegradation pathways,^14−16^ and biological information.^17−19^ Studies that have employed invasive sampling techniques favor these themes too, with investigations typically exploring parchment degradation by microbial or environmental action.^20−24^ The value of minimally invasive or conservation-aligned analysis is witnessed by the growth of biocodicology. In this study, the term “minimally invasive” is used in accordance with conservation science principles and denotes sample removal that has only minimal impact on the material integrity, such as the removal of a small sample, or in our case, sample removal at the molecular level. Invasive sampling hence describes any physical removal of a sample.^25^

The production of parchment induces several changes in the structural composition of animal skin, with the finished product primarily comprising the dermis layer (ca. 95% collagen).^26^ During the liming stage of production, amide side chains from proteins and triacylglycerols from subcutaneous lipids both located in the animal dermis and the subcutaneous layer undergo partial base-hydrolysis. The alkaline conditions during this stage also facilitate the cleavage of disulfide bonds in keratin, aiding in the dehairing process.^27−30^ The removal of residual epidermal tissues during fleshing, washing, and smoothing results in further reduction of the lipid content to only a minor fraction; this is crucial to whiten the material and improve its capacity to absorb inks.^5^

The presence of a lipid fraction has previously been reported in historical parchment by gas chromatography–mass spectrometry (GC-MS), small-angle X-ray scattering, and solid-state NMR.^31^ Here, a minor lipid component containing saturated and monounsaturated fatty acids (C_14_–C_24_), cholesterol, and triacylglycerols was identified as reflecting the typical profile of degraded animal fats.^32^ The origin of this fraction was initially attributed to either the incomplete removal of dermal fats during the manufacturing process (endogenous) or attempts at conservation, handling, or microbial attack of collagen which introduced lipids over time (exogenous).^31^ It has since been suggested that endogenous lipids are more likely to contribute to the detectable lipid fraction thus reflecting the original characteristics of the animal it was made from.^33^ Studies have also investigated the lipid fraction within parchment by multianalytical techniques^34,35^ but primarily report the role of lipids in terms of their effect on the degradation of parchment and its conservation.

Stable isotope analysis of collagen extracted from parchment has emerged as a potential tool for exploring changes in agricultural strategies, dietary reconstructions, and parchment production using the unique specificity that each parchment folio corresponds to an individual animal.^5,36,37^ However, conducting such studies necessitates the use of invasive sampling techniques to obtain the required milligram quantities of collagen;^37^ studies are consequently constrained to sampling parchment of little historic value or require sacrificial parchment to be manufactured.^5^ Given that lipids are ^13^C-depleted relative to proteins,^38^ isotope studies of parchment include a “defatting” step, in which the lipid component is removed using organic solvents and usually discarded. However, animal fats extracted from ancient matrices are recognized as being recorders of metabolic (ruminants vs nonruminants), dietary, and climate information through their stable isotope compositions.^39−41^ Herein, we acknowledge that the determination of carbon isotope composition of lipids extracted from parchment could serve as a complementary approach to collagen analysis, enabling metabolic and dietary information to be derived from individual membranes reflecting the lives of individual animals.

Until now, the solvent extraction of parchment lipids has only been achieved by invasive methods requiring sacrificial fragments, in some cases, ca. 7.5–20 cm^2^ of material.^31^ As a result, this type of sampling is unsuitable for historically valuable documents and can present a considerable analytical challenge even for nonvaluable parchment. Here we propose a minimally invasive lipid extraction technique aided by a vacuum. A sampling table and two glassware components that comprise a flat, fritted face funnel and collection vial of a similar design to a boiling tube were bespoke designed (Figures 1 and S1). To perform the extraction, the face of the funnel is placed underneath the surface of the parchment, and while under vacuum, a solvent mixture (DCM/MeOH, 2:1, v/v) is added dropwise to the reverse face of the material. The solvent is drawn through the parchment membrane and into the collection vial before analysis by chromatographic and mass spectrometric techniques (S2).

**Figure 1:**
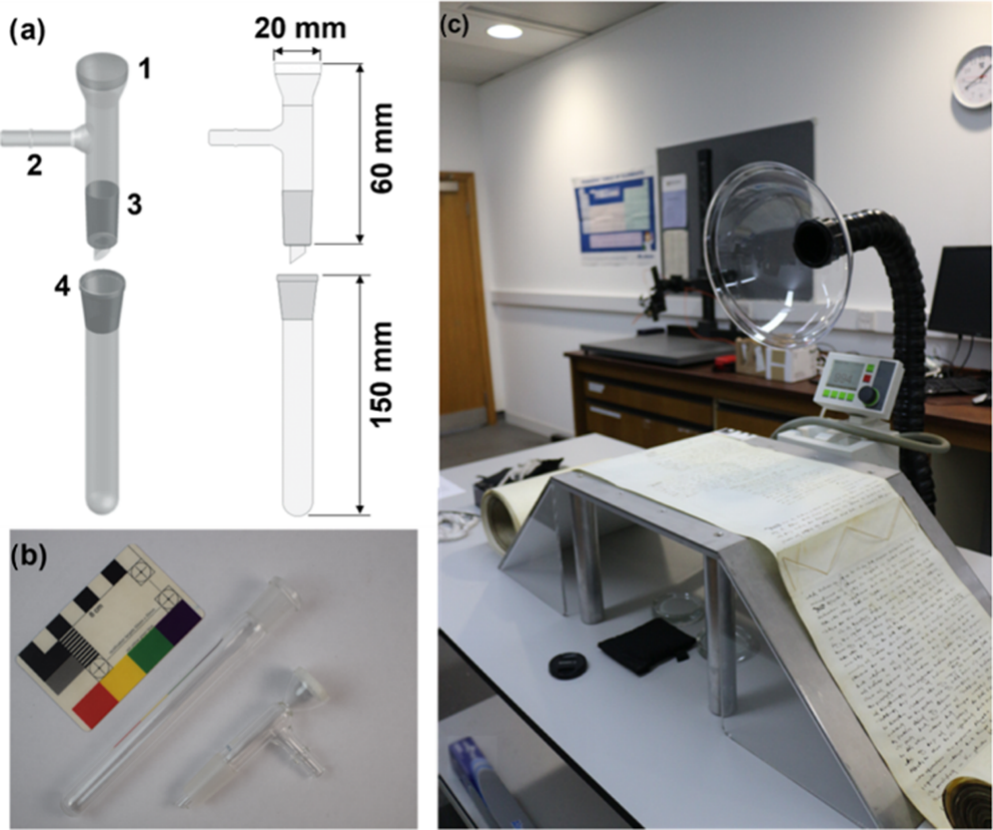
(a) Schematic diagram of the vacuum-aided extraction glassware apparatus where (1) indicates a flat, fritted face; (2) an arm connecting to the vacuum source; (3) the connecting joint between the funnel and collection vial; and (4) the collection tube. (b) Image of the funnel and collection vial disassembled. (c) Image of the sampling setup at The National Archives. The Roll is placed on an aluminum sampling table pierced with 5 cm-diameter holes to enable the face of the glass funnel to be placed underneath the parchment. An extraction unit was used to avoid solvent vapors. Photo credit: Samuel P. Johns.

We first investigated the suitability of the vacuum-aided extraction method as an alternative for the invasive sampling of parchment by comparing the quantitative and qualitative compositions of lipids recovered from nine sacrificial parchments. The parchments comprised British legal deeds of unknown history and were dated 1765–1825 CE (Table S1). The recovery and molecular composition of lipid extracts obtained by the novel technique was compared to invasive extracts taken from the same sample area (<1 cm distance). Parchment heterogeneity was then investigated by studying samples obtained using vacuum-aided extraction from different regions of the sacrificial parchments. Having established that the vacuum-aided extraction had no immediate or long-term effects to the material,^42^ lipids from membranes of the Chancery Parliament Rolls (Table S2) were extracted and their molecular and isotope compositions determined to infer taxonomic and dietary information. This information was then compared with results from peptide mass fingerprinting (ZooMS) for species identification.^17,18^

## Materials and Methods

### Sacrificial Samples

Nine sacrificial parchment legal deeds were obtained from the Beast2Craft Collection^43^ (Table S1). The history and storage conditions were unknown, but parchments were generally well-preserved, with few visual signs of degradation. One manuscript (a 1790 CE division of an estate) evidenced some water damage. The 1786 CE indenture comprised three parchment manuscripts which were labeled 1786a–c.

### Chancery Rolls—UK Acts of Parliament

Chancery Rolls^44^ documenting UK Acts of Parliament were obtained from The National Archives. Rolls of parchment comprised tens of membranes sewn together. Five membranes from five different Rolls were sampled for each of the seven years (*n* = 5) except for when fewer than five Rolls per year were available (year 1820). In this case, extractions were performed on different membranes from the same Roll (Table S2). Extractions were taken from regions avoiding any ink, writing, or visible soiling, typically within the left-hand margin ca. 5 cm from the edge of the membrane.

### Sampling Methods

#### Glassware, Solvents, and Reagents

Reusable glassware was cleaned with Decon-90 (Decon Laboratories) and then rinsed with acetone before oven-drying and furnacing (450 °C; 4 h); disposable glassware was also furnaced (450 °C; 4 h). HPLC grade solvents (≥99% purity; Fisher Scientific/Rathburn) and analytical grade reagents (≥98% purity; Fisher Scientific/Rathburn) were used for all experimentation.

#### Invasive Sampling

A 3 × 1 cm^2^ sample of parchment was removed and split into three 1 × 1 cm^2^ squares using a solvent-cleaned scalpel from each of the nine sacrificial manuscripts and placed in a 7 mL vial containing 10 μg of *n*-tetratriacontane (C_34_) and 10 μg of heneicosanoic acid (C_21:0_).

#### Vacuum-Aided Extraction Sampling

Parchments were elevated from the workbench using an in-house designed sampling table comprised of a stainless-steel fascia. The face of the table comprised several holes of 5 cm diameter to enable the sampling of manuscripts (Figure S1). A BUCHI Vacuum Pump V-700 and BUCHI Vacuum Controller V-850 were connected to an in-house-constructed vacuum funnel featuring a detachable vial (Figure 1a,b). A DCM/MeOH mixture (2:1, v/v, 1 mL) was added to the glass frit of the vacuum funnel before applying a vacuum of ca. 700–850 mbar. Upon contact between the manuscript and funnel, DCM/MeOH (2:1 v/v; 3 mL) was pipetted dropwise to the reverse side of the manuscript, where it was drawn through the manuscript by the vacuum. Following extraction, the funnel was removed from the manuscript and vacuum paused, a further 3 mL of DCM/MeOH (2:1 v/v) was used to rinse the glassware frit; the contents of the extraction and solvent rinse were collected in the detachable vial containing 10 μg of *n*-tetratriacontane (C_34_) and 10 μg of heneicosanoic acid (C_21:0_). A video of the extraction technique has been made available at https://drive.google.com/file/d/1htWy-LcbJiJHYAzqAHWP3eVgpY3BQ6r3/view?usp=drive_link.

#### Protein Extraction

Eraser rubbings (ca. 50 μL) were taken from parchments following existing protocols^18^ ensuring to avoid areas of visual deterioration, ink, or soiling (S3–S4).

### Sample Preparation

#### Lipids

Total lipid extracts of vacuum and invasive reference samples were assessed in accordance with established protocols.^45,46^ An aliquot taken from each total lipid extract (TLE) was derivatized using *N*,*O*-bis(trimethylsilyl)trifluoroacetamide containing 1% trimethylchlorosilane before analyzing by HTGC-FID and HTGC/MS. A second aliquot was saponified (NaOH/MeOH, 9:1, v/v; 70 °C, 1 h) and methylated (BF_3_/MeOH, 14% w/v; 70 °C, 1 h) and used for the determination of total fatty acid content.

#### Proteins

Eraser rubbings obtained from parchments were prepared according to established protocols (S4).^18^ Samples were generated by extracting degraded collagen, which was gelatinized prior to enzyme treatment with porcine trypsin.

### Instrumentation

Trimethylsylilated (TMS) and methylated total lipid extracts were screened and quantified using HTGC-FID and HTGC-MS. The δ^13^C values of fatty acid methyl esters were determined using GC-C-IRMS. Protein samples were analyzed by MALDI-TOF. See S2–S4 for details about instrumentation.

## Results and Discussion

### Lipid Recovery with Vacuum-Aided vs Invasive Extraction

**Figure 2:**
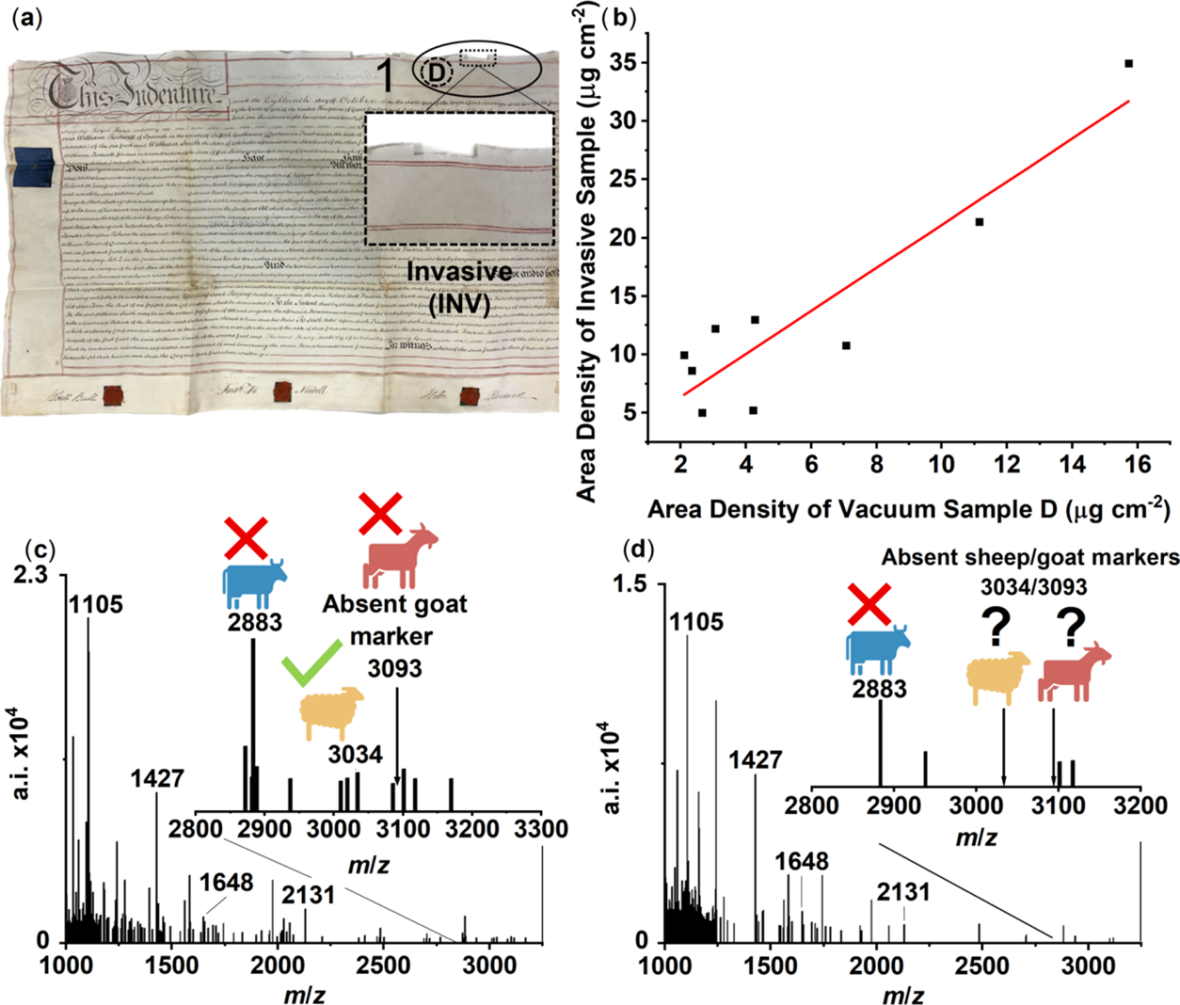
(a) Representative sampling scheme applied to each of the nine sacrificial parchments dated 1765–1825 CE. The approximate location of vacuum samples is depicted by the dashed circle (D) and occurs along the horizontal edge (Region 1). An invasive sample (INV) was taken close to sample D on the horizontal edge of the parchment. Photo credit: Samuel P. Johns. (b) Comparison of the total fatty acid area density (μg cm^–2^) of the vacuum-aided and invasive samples taken from the horizontal edge (Region 1) of each of the nine parchments dated 1765–1825 CE, with *y* = 2.68 + 1.84*x* and *R*^2^ = 0.85. (c, d) Peptide mass fingerprint for two sacrificial parchments (legal deeds) dated 1816 and 1786 CE, respectively, obtained using triboelectric sampling and ZooMS analysis. Peptide markers at *m*/*z* 2883 were used to exclude the presence of cow species. Sheepskin was identified through the presence of *m*/*z* 3034 peptide markers and the absence of goat makers at *m*/*z* 3093 (c). In the absence of peptide markers diagnostic of sheep or goat, species could not be distinguished (d).

To evaluate the efficacy of the vacuum-aided extraction to recover lipids from parchment compared to conventional, invasive sampling, closely located vacuum-aided and invasive (three 1 × 1 cm^2^) samples were taken from nine sacrificial parchments dated 1765–1825 CE. Both samples were obtained from neighboring areas along the same horizontal edge of the parchment (Region 1, Figure 2a), avoiding areas of writing or visual soiling. Fatty acid methyl esters (FAMEs) were prepared by base-hydrolysis and methylation of the respective total lipid extracts before quantification by high-temperature gas chromatography with a flame ionization detector (HTGC-FID) against an internal standard added at the time of extraction.

The total fatty acid area density, expressed in μg of lipids per cm^2^ of parchment, of the vacuum-aided edge samples follows a linear relationship with that of the invasive edge samples (Figure 2b). The total fatty acid area densities from vacuum-aided horizontal edge samples were significantly different from that of the invasive edge samples taken from neighboring areas (Mann–Whitney *U* test, *Z* = −2.3, *p* = 0.02, Table S5) with the vacuum-aided extraction recovering ca. half the quantity of lipids compared to the invasive technique. The lipid recovery between parchments varies considerably with a range of 2.1–15.7 and 5.0–35.5 μg cm^–2^ for the vacuum-aided and invasive samples, respectively.

While there has been little investigation comparing the lipid content of parchment from different animal species, the differences between animal species and breed skins are well-documented through histological studies.^47^ A recent study on 16th–20th-century British legal deeds using peptide mass fingerprinting (ZooMS) showed that 96.4% of the parchments were made from sheepskin (*Ovis aries*), due to both the availability of sheepskin and its distinctive structure.^48^ Sheepskin, unlike goat and calfskin,^18^ contains a secondary lipid layer which lies separate from the subcutaneous fat.^47^ As a result, the lipid content of sheepskin range between 30 and 50% of the dermis dry weight, as opposed to 2–3 and 3–10% for cattle and goatskin, respectively;^18,48−51^ sheepskin is noted as more challenging to work with by parchment makers due to its higher lipid content.^52^

To determine the effect of animal species in this study, each of the nine parchments were triboelectrically sampled and species identified using ZooMS (S4) as described elsewhere.^19^ All nine parchment samples featured peptide markers at *m*/*z* 2883 (therefore excluding cattle). Six parchments were identified as sheepskin due to peptide markers at *m*/*z* 3034 (diagnostic of sheep, *Ovis aries*) and absent *m*/*z* 3093 (diagnostic of goat, *Capra hircus*). Three parchments were identified as sheep/goat due to the absence of diagnostic peptide markers at *m*/*z* 3034 (sheep) or *m*/*z* 3093 (goat; Table S3, Figure 2c,d).^53^ In spite of this, no British legal deed has ever been identified as goatskin by peptide mass fingerprinting;^18,37,48^ therefore, it is reasonable to assume that all of these parchments were made from sheepskin (as first directed by Richard Fitznele)^54^ and that the variability in fatty acid area density is due to a factor other than differing animal species.

### Molecular Composition of Lipid Extracts

**Figure 3:**
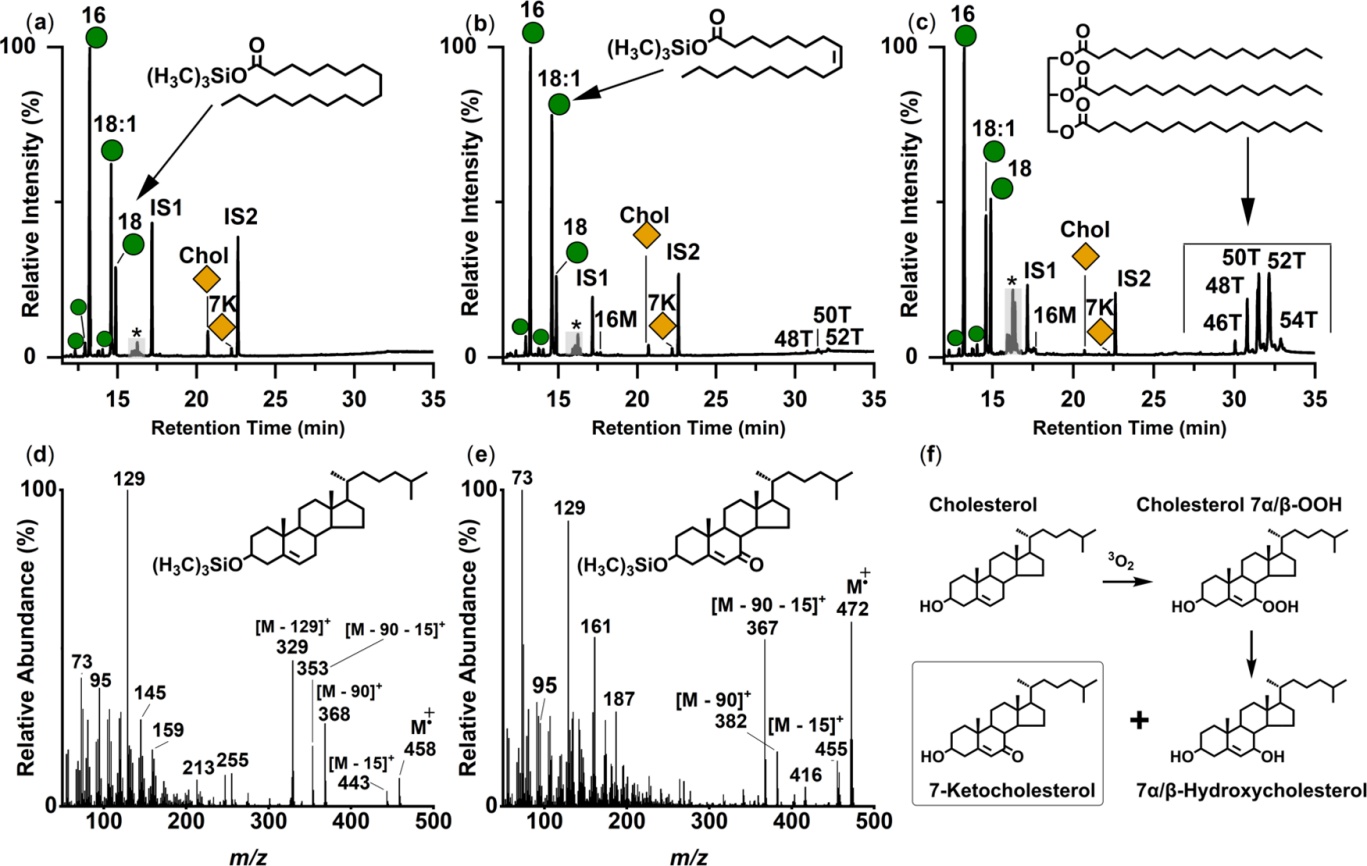
Typical partial total ion current chromatograms of TMS-derivatized total lipid extracts obtained from a sacrificial parchment (1817 CE) via (a) invasive sampling and (b) vacuum-aided extraction of neighboring areas (parchment edge, Region 1); and (c), vacuum-aided extraction of the parchment center (Region 3, Figure 4a). Green circles indicate fatty acids; orange diamonds, sterols; IS1-IS2 internal standard (heneicosanoic acid and *n*-tetratriacontane, respectively). Numbers *n* and *n:i*, acyl carbon number with zero or *i* degrees of unsaturations. *n*M and *n*T are mono- and triacylglycerols (TAGs) containing *n* number of carbon atoms; Chol: cholesterol, 7K: 7-ketocholesterol. Black asterisks mark unidentified compounds. Mass spectra of TMS-derivatized cholesterol (d) and 7-ketocholesterol (e), respectively. (f) Free radical-mediated oxidation pathway of cholesterol to 7-ketocholesterol and 7-hydroxycholesterol. Adapted from Int. J. Biochem. Cell Biol., Vol. 31, Lyons, M. A.; Brown, A. J. 7-Ketocholesterol, pp. 369–372 (ref (61)). Copyright 1999, with permission from Elsevier.

Total lipid extracts obtained from both the invasive and vacuum-aided extractions of sacrificial parchments were trimethylsylilated and analyzed by HTGC-FID and HTGC-MS. Chromatograms display lipid profiles comprising free saturated and unsaturated *n*-alkanoic acids (dominated by C_16:0_, C_18:1_, and C_18:0_; Figure 3a–c) consistent with degraded animal fats.^32,55^ No or little evidence of triacylglycerols was detected in the extracts. Crucially, the extracts obtained using the vacuum-aided method from areas located next to the invasive samples (Region 1; Figure 3a,b) display a similar lipid profile in all nine parchments suggesting that the type of extraction does not affect the lipid composition. To assess the consistency of this lipid profile, a separate vacuum extraction of the center region was evaluated (Region 3, Figure 3c) and identified TAGs (C_46_ – C_54_). Cholesterol was identified in all samples (Figure 3d). As the main component of mammalian cell membranes and a precursor for steroid-based hormones, its presence likely arises from endogenous origin.^56^ However, owing to the potential for exogenous contamination to occur through the handling of parchment over time, its origin should be treated with caution. The absence of squalene, a human steroidal precursor also found in skin,^57^ in conjunction with the presence of its characteristic degradation products such as 7-ketocholesterol (Figure 3e),^56,57^ provides good evidence for endogenous cholesterol. While 7-ketocholesterol has been studied in degradation experiments of cholesterol,^56,58^ it has not been identified in published literature of parchment or fresh sheepskins.^59,60^ 7-Hydroxycholesterol is reported as an oxidation product formed via the same free radical-mediated degradation pathway as 7-ketocholesterol (Figure 3f) but was not identified in parchment samples. This is likely due to the preferential formation of 7-ketocholesterol during oxidation.^61^

To ascertain the endogeny of 7-ketocholesterol, solvent extractions of three fresh sheepskins and newly manufactured parchments derived from the same skin were performed. 7-Ketocholesterol was identified in all extracts, and its abundance was compared to that of cholesterol. The ratio of cholesterol to 7-ketocholesterol was higher in skin than in newly manufactured parchment (52 and 23, respectively). The same ratio was determined from the historic parchment, which was an order of magnitude lower (3.4). Given that cholesterol oxidation products are formed exclusively from cholesterol and influenced by pro-oxidation agents such as temperature, light, oxygen, and moisture,^62^ it is likely that cholesterol undergoes oxidation to form 7-ketocholesterol in parchment following its manufacture and storage over time. This transformation would lead to a decrease in the concentration of cholesterol relative to 7-ketocholesterol and may explain why historic samples evidenced lower ratios than both skin and freshly manufactured parchment.

### Lipid Heterogeneity within the Same Parchment

**Figure 4:**
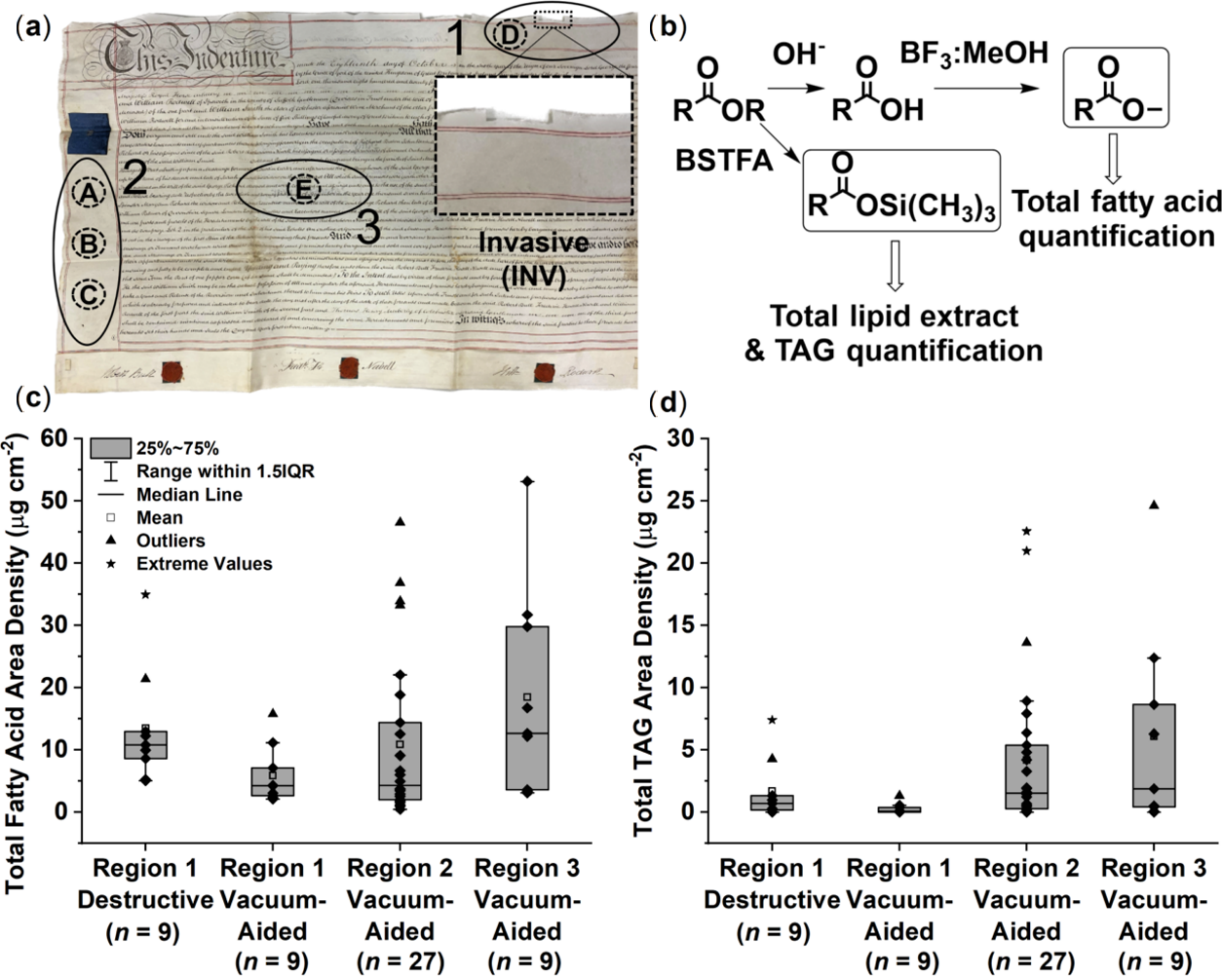
(a) Representative sampling scheme applied to each of the nine sacrificial parchments dated 1765–1825 CE. The approximate location of vacuum samples is depicted by dashed circles (A–E) and occurs within three regions: (1) horizontal edge, (2) vertical margin, and (3) center. An invasive sample (INV) was taken close to sample D on the horizontal edge of the parchment. Photo credit: Samuel P. Johns. (b) Pathway scheme for the derivatization and quantification of total lipid extracts and total fatty acids. (c, d) Box-and-whisker plots showing the total fatty acid (c) and TAG (d) area densities (μg cm^–2^) within the nine sacrificial parchments. The edge region (Region 1) comprised a single vacuum-aided and invasive extraction; the vertical margin region (Region 2) three vacuum-aided extractions (A–C), and the center region (Region 3) a single vacuum-aided extraction. The horizontal line within each box indicates the median value area density and the square data plot indicates the mean average value. The lower and upper boundaries of the boxes indicate the 25th and 75th percentiles, respectively. Data points are shown as diamonds, while outliers and extreme outliers are identified as triangular and asterisk data plots greater than 1.5× and 3× the interquartile range, respectively. Whiskers above and below the boxes extend to the largest and smallest values within 1.5× the interquartile range.

Heterogeneity of animal skin is acknowledged as a considerable analytical challenge in many noninvasive studies of parchment requiring repeat measurements or multiple samples.^63^ Indeed the heterogeneity in the lipid makeup in animal skin and, or otherwise, the heterogeneous removal of lipids during the manufacturing process are both affecting the lipid content of parchment. Lipid heterogeneity was investigated using further vacuum-aided extractions taken from the left-hand, vertical margin of the nine sacrificial parchments, a few cm away from the edge (extraction A-C, Region 2; Figure 4a) and in the center of the parchment (extraction E, Region 3; Figure 4a). While it has previously been identified that the thickness of parchment is likely inhomogeneous,^22^ the locations of vacuum extractions precluded comparable measurements in this study. The total lipid extracts were base-hydrolyzed and methylated to determine the total fatty acid content for each of the extractions while the TAG area density and molecular composition of the extracts was determined by HTGC-MS analysis of the total lipid extracts after trimethylsylilation (Figure 4b–d).

The total fatty acid area density of vacuum-aided extractions from the center region (Region 3, E; median: 12.7 μg cm^–2^, σ = 15.9 μg cm^–2^) was significantly higher (*Z* = −2.1, *p* = 0.04; Table S6) than extracts from the vacuum-aided horizontal edge (Region 1, D; median: 4.2 μg cm^–2^, σ = 4.4 μg cm^–2^; Figure 4c). Similarly, the center region evidenced a greater fatty acid content than the vertical margin (Region 2, A–C; median: 4.3 μg cm^–2^, σ = 12.7 μg cm^–2^) although this was not significant (*Z* = −1.5, *p* = 0.13; Table S7). The lipid content is typically greater in regions further from the edges of parchment with the center region containing twice the area density than the edges. The optimal sampling location for lipid recovery is thus the center part of parchments, containing higher concentrations of lipids. However, the use of organic solvents in the vacuum-aided extraction has the potential to solvate and/or damage inks,^64^ and thus this location may be not accessible for use on documents of historic value. Because the vacuum-aided extraction offers the optional benefit of performing multiple extractions on the same membrane, higher amounts of lipids can be recovered when performing three consecutive extractions (Region 2, A–C; combined: median: 187 μg, S7, Figure S2) than would be possible by invasive sampling (25 μg; 1 cm^2^).

The molecular composition of extracts obtained through vacuum extraction was compared across different regions of the parchment. Triacylglycerols (TAGs) were typically absent or in low quantities in the edge samples (Region 1, 0.0–1.3 μg cm^–2^), while TAGs C_44_–C_54_ were detected in the center vacuum extraction (Region 3) at high concentrations (0.0–24.6 μg cm^–2^) and in the vertical margin (Region 2) for most parchments (0.0–22.5 μg cm^–2^; Figure 4d). In the edge extractions, TAGs have likely undergone complete hydrolysis before the time of analysis.^65^ The absence of mono- and diacylglycerols is likely explained by the increased tendency for acyl lipids to undergo complete hydrolysis following the loss of a single fatty acid moiety.^66^ Parchment is susceptible to degradation following its manufacture by a combination of factors including microbial action, humidity, atmospheric pollutants, and temperature.^23,67^ Collagen at the edges of parchment was also shown to be more degraded than in central areas; this was attributed to the edges being more exposed to the degradative process through time.^35^ Both lipids and collagen are thus likely to be prone to degradation processes at the exposed edge regions of parchments rather than in the center regions. The abundance of 7-ketocholesterol further corroborated results of TAG hydrolysis, with greater area densities observed in Region 1 (invasive: 0.3–1.5 μg cm^–2^; vacuum-aided D: 0.0–0.2 μg cm^–2^) than in Region 2 (0.0–0.2 μg cm^–2^) and Region 3 (0.0–0.2 μg cm^–2^). Finally, the handling of documents at the corners and edges may introduce exogenous lipids,^31^ but no anthropogenic markers were detected in the extracts (squalene, vitamin E).^57^

### Molecular and Isotope Composition of Membranes from the Parliament Rolls

The vacuum-aided extraction was shown to provide a robust method for the minimally invasive extraction of lipids from parchment. Using this method on historic nonaccessioned parchments, Vermeulen et al. demonstrated using a multianalytical approach that extracted areas of parchment did not present any immediate or long-term changes to the integrity of the material.^42^

**Figure 5:**
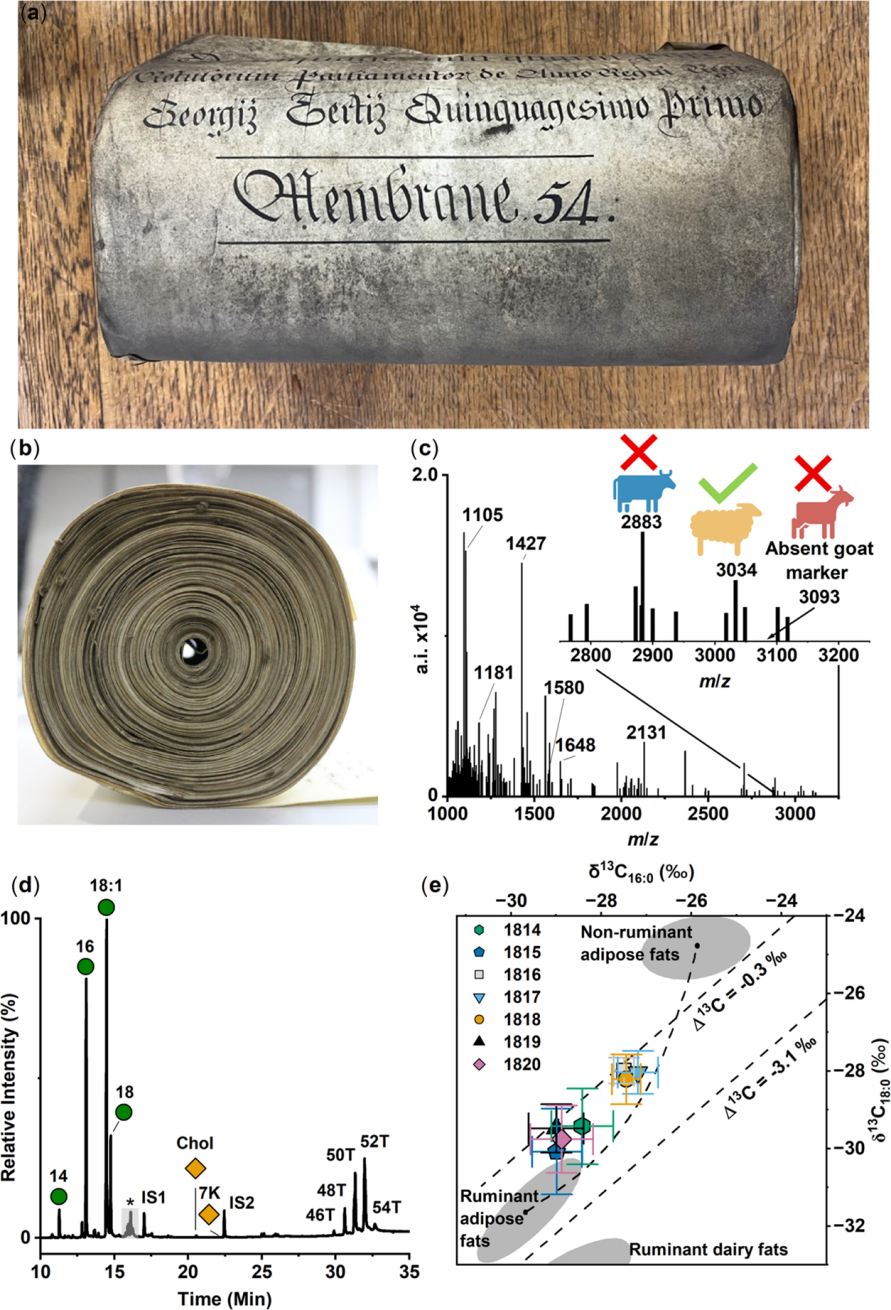
(a, b) Parliament Roll (C65) from The National Archives collection (with permission; top and side view, respectively). Photo credit: Melanie Roffet-Salque. (c) Representative peptide mass spectral fingerprint concerning the Parliament Roll (1814 CE) obtained using triboelectric sampling and ZooMS analysis. Parliament Rolls were identified as sheepskin based on diagnostic peptide markers (*m*/*z* 3034: sheep; *m*/*z* 2883: not cattle) and the absence of goatskin peptide markers (*m*/*z* 3093). (d) Typical partial total ion current chromatogram of a TMS-derivatized total lipid extract from a Parliament Roll (1819 CE). Green circles indicate fatty acids; orange diamonds, sterols; IS1-IS2 internal standard (heneicosanoic acid and *n*-tetratriacontane, respectively). Number *n* and *n:i*, acyl carbon number with zero or i degrees of unsaturation. *n*T are triacylglycerols (TAGs) containing *n* number of carbon atoms; Chol: cholesterol, 7K: 7-ketocholesterol. Black asterisks mark unidentified compounds. (e) Mean average δ^13^C values (*n* = 5) of C_16:0_ and C_18:0_ fatty acids extracted from Parliament Rolls (1814–1820 CE). Ellipses and ranges are indicative of modern reference fats (*P* = 0.68 confidence ellipse) from animals raised on C_3_ diets. Δ^13^C = δ^13^C_18:0_ – δ^13^C_16:0_. Data used with permission from Proceedings of the National Academy of Sciences USA Copley, M. S.; Berstan, R.; Dudd, S. N.; Docherty, G.; Mukherjee, A. J.; Straker, V.; Payne, S.; Evershed, R. P. J. Proc. Natl. Acad. Sci. U.S.A. 2003, (4), 100, 1524–1529 (ref (39)). Error bars in the *X* and *Y* directions are representative of ±1σ.

We then turned to the Chancery Parliament Rolls documenting UK Acts of Parliament to demonstrate the applicability of lipid analysis on valuable historic parchments with well-documented histories. These prestigious documents represent one of the longest-running parchment collections in the UK (ca. 1427–2010 CE). The series is made up of records of parliamentary proceedings (until 1483), then increasingly from the 15th century onward copies of Acts of Parliament which received Royal Assent.^44^ Much like other parchment, these documents were used within short succession of animal slaughter.^68^ A limited number of trusted parchment makers were responsible for supplying parchment for the Parliament Rolls and thus their membranes have an origin traceable to South Central England.^68^ After writing, the Rolls were stored and handled thereafter; however, such occurrences were infrequent, as demonstrated in reports of the Deputy Keeper of the Public Records, revealing only two documented searches of Parliament Rolls in the year 1837.^69^ A total of 35 parchments, dated 1814–1820 CE (*n* = 5 per year), from the Chancery Parliament Rolls collection (Figure 5a,b) were sampled on areas free from writing using the vacuum-aided extraction method.

To ascertain the taxonomic origins of the Parliamentary parchment Rolls, collagen in triboelectric samples was analyzed by ZooMS.^19^ All parchment samples evidenced peptide markers at *m*/*z* 2883 (excluding cattle) and *m*/*z* 3034 (diagnostic of sheep), with an absence of *m*/*z* 3093 (diagnostic of goat), ascertaining the origins of the skins to sheep, a ruminant animal (Figure 5c, Table S4).^53^ The Chancery Parliament Rolls represent a remarkable record of sheepskins, the animal whose wool in 1353 CE is according to the Ordinance of the Staple “the sovereign merchandise and jewel of this realm of England”,^70^ and which fills the sacks upon which the Lord Chancellor sits in the House of Lords.

The molecular composition of the extracts is typical of degraded animal fats,^32,55^ but, unlike the sacrificial parchments, triacylglycerols were in high abundance echoing the overall good preservation of the Rolls (Figure 5d). Fatty acid methyl esters were prepared from the total lipid extracts and the δ^13^C values of C_16:0_ and C_18:0_ alkanoic acids determined by GC-C-IRMS (Figure 5e). All samples exhibit Δ^13^C (= δ^13^C_18:0_ – δ^13^C_16:0_) values ranging from −1.1 to −0.5‰ and are consistent with adipose fats from ruminant animals (e.g., sheep, goats, cattle).^39^ Extractions of membranes from the same year display very close δ^13^C values (1σ: ± 0.1 to ±1.1‰). The δ^13^C values of C_16:0_ fatty acids from the extracted skin fats range from −29.0 to −27.2‰ and are consistent with adipose fats from ruminants raised on a C_3_ or mixed C_3_/C_4_ diet.^40^ Collagen samples from British parchments display higher δ^13^C values following the Napoleonic Wars (1803–1815 CE), attributed to transformations in both animal husbandry and land management practices, which led to an increased consumption of oil-cake fodder, primarily derived from imported crops such as C_4_ maize.^37^ The δ^13^C values obtained from lipids extracted from the Parliamentary Rolls over 1814–1820 CE are thus reflecting the source (a ruminant animal, sheep) and changes in animal feeds.

## Conclusions

This study investigated the capacity for a minimally invasive vacuum-aided method to recover lipids from historical parchments. The novel extraction technique yielded ca. half the quantity of lipids compared with invasive sampling, a quantity suitable for the determination of compound-specific stable carbon isotope compositions from parchment. Gas chromatographic and mass spectrometric analyses indicated similar lipid profiles in both vacuum-aided and invasive samples, affirming that the extraction method did not influence the molecular composition. Notably, 7-ketocholesterol was identified in parchment for the first time, providing evidence of cholesterol oxidation over time. The heterogeneity of parchment was investigated through quantitative assessment of vacuum and invasive extractions from different regions of the same parchments. Extractions of the central region were found to demonstrate typically higher area densities of total fatty acid attributed to nonuniform exposure to lipid degradation factors. By combining three vacuum-obtained extracts from the same region, we designed an optimal sampling scenario which mitigates the risk of damaging inks and recovers over twice the median average fatty acid content when compared to invasive sampling.

This vacuum-aided extraction method for the minimally invasive extraction of lipids from parchments was applied for the first time to valuable parchments from The National Archives (UK) collections. Lipids were successfully extracted from various membranes of the Chancery Parliament Rolls. The carbon isotope compositions of the extracts reflect the lipid source (ruminant fats) and shed light on animal diets. Collagen fingerprinting (ZooMS) allowed us to conclusively identify membranes from the Chancery Parliament Rolls as being derived from sheepskin. This novel approach aids reconstructions of animal diet and parchment taxonomic origin, paving the way for further molecular and isotope analysis of well-dated, historically valuable parchments for the reconstruction of paleoclimate. While the application of this technique has focused on the extraction of parchment lipids in this study, we anticipate that our vacuum extraction method may be suitable for extracting organic compounds from other ancient materials, such as fabric and paper.

## Supporting Information Available

S2 further materials and methods; S4 detailed results of peptide mass fingerprinting; S6 statistical analysis; S7 lipid heterogeneity within the same parchment; S8 references (PDF)
