# Supplementary material for: Minimally Invasive Vacuum-Aided Extraction Technique for the Lipid Analysis of Historic Parchment
Source: Anal Chem. 2024 Aug 16;96(34):13811–20. doi: 10.1021/acs.analchem.4c01395 (PMC11359388; doi:10.1021/acs.analchem.4c01395)
Supplement: Supplementary file 1 — ac4c01395_si_001.pdf [file ac4c01395_si_001.pdf]

Supporting Information

Minimally Invasive Vacuum-Aided Extraction Technique for the Lipid Analysis of Historic Parchment.

Samuel P. Johns[1*], Charlie A. Maule[1], Lora Angelova[2], Marc Vermeulen[2], Chris Day[3], Marta Muñoz-Alegre[4], Matthew J. Collins[4,5], and Mélanie Roffet-Salque[1*]

1. Organic Geochemistry Unit, School of Chemistry, University of Bristol, Cantock's Close, Bristol, BS8 1TS (UK).
2. Collection Care Department, The National Archives, Bessant Drive, Richmond, London, TW9 4DU (UK)
3. Collection Expertise and Engagement Department, The National Archives, Bessant Drive, Richmond, London, TW9 4DU (UK)
4. McDonald Institute for Archaeological Research, University of Cambridge, Downing Street, Cambridge, CB2 3ER (UK)
5. The Globe Institute, University of Copenhagen, Oster Voldgade 5-7, 1353 Copenhagen K (DK)

* Corresponding authors: Email: samuel@palaeome.org and melanie.salque@bristol.ac.uk

Table of Content

Materials and methods S2
Peptide mass fingerprinting S4
Statistical analysis S6
Lipid heterogeneity within the same parchment S7
References S8

Materials and methods

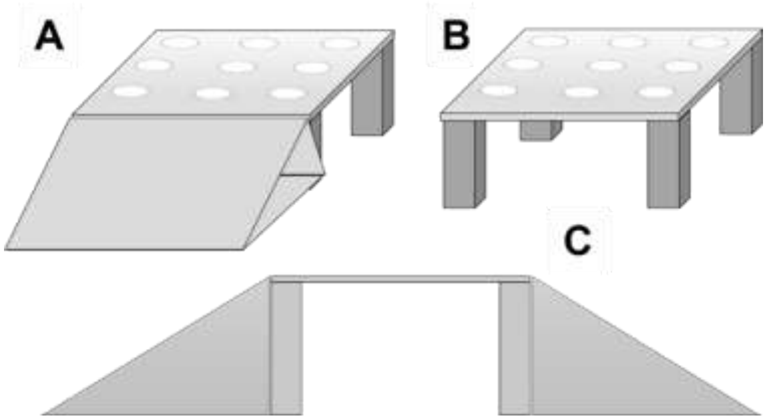

Figure S1: Illustration of sampling table designed by the authors for the vacuum extraction of Parliamentary Rolls. Face of sampling table and 1/2 ramps (A). Free-standing sampling table (B). Side profile of sampling table and 2/2 ramps (C). Parchment rolls may be unwound using the ramp system and samples taken using the holes in the table.

Table S1: Sacrificial parchment samples obtained from the Beast2Craft collection.[1]

Date/ CE	Details	Location	Dimensions (*l* x *h*)/ cm
1765	Indenture	Liverpool	85 x 64
1786a	Mortgage	Bristol	78 x 62
1786b	Mortgage	Bristol	78 x 62
1786c	Mortgage	Bristol	78 x 62
1790	Division of Estate	Widnes	72 x 62
1810	Lease	Peasmarsh	84 x 56
1816	Mortgage	Hereford	86 x 70
1817	Mortgage	Strand, London	84 x 52
1825	Lease	Bermondsey, London	62 x 38

Table S2: Chancery Parliamentary Rolls obtained from The National Archives listed for each year. Roll identifications correspond to official Acts of Parliament accessed via The National Archives.[2] Asterisk indicates multiple sampling from the same roll but different membranes.

Date/ CE	Roll ID 1	Roll ID 2	Roll ID 3	Roll ID 4	Roll ID 5
1814	C 65/2218	C 65/2230	C 65/2242	C 65/2254	C 65/2266
1815	C 65/2266	C 65/2288	C 65/2298	C 65/2308	C 65/2318
1816	C 65/2338	C 65/2348	C 65/2349	C 65/2350	C 65/2360
1817	C 65/2378	C 65/2379	C 65/2381	C 65/2381	C 65/2410
1818	C 652411	C 65/2421	C 65/2425	C 65/2432	C 65/2438
1819	C 65/2452	C 65/2473	C 65/2479	C 65/2491	C 65/2503
1820	C 65/2517*	C 65/2517*	C 65/2517*	C 65/2518*	C 65/2518*

HTGC-FID

Trimethylsylilated (TMS) and methylated total lipid extracts (TLEs) were screened and quantified using an Agilent Technologies 7890A GC gas chromatograph equipped with a fused silica capillary column (15 m x 0.32 mm) coated with dimethylpolysiloxane stationary phase (DB-1HT; film thickness, 0.1 µm; Agilent Technologies). Derivatized TLE extracts were injected on-column at an oven temperature of 50 °C (2 min, isothermal hold) before increasing to 350 °C (10 °C min^{-1} ramp; 10 min^{-1}, isothermal hold). Helium was used as the carrier gas (flow rate: 4 mL min^{-1}). The temperature of the flame ionizing detector (FID) was set to 350 °C. Quantification was performed through the addition of internal standards (C_{34}, *n*-tetratriacontane; $C_{21:0}$, heneicosanoic acid) during the sample preparation stage. Data acquisition and processing were conducted using Agilent MSD ChemStation software (F.01.01.2317, Agilent Technologies).

HTGC-MS

HTGC-MS analyses of the TMS-derivatized TLE sample diluted in ca. 50 µL of hexane was performed using a Thermo Scientific Trace 1300 gas chromatograph equipped with a 15 m × 0.32 mm i.d. fused-silica capillary column coated with dimethyl polysiloxane stationary phase (Rxi-1HT; film thickness, 0.1 µm; Restek); this was coupled with a Thermo Scientific ISQ single quadrupole mass spectrometer. A total of 1 µL of the sample was introduced using a PTV injector in split mode (split flow 30 mL min^{-1} split ratio 6.0). The oven temperature was isothermally held for 2 min at 50 °C before increasing to 280 °C (10 °C min^{-1} ramp) then at a rate of 25 °C min^{-1} to 380 °C; this was held isothermally for 5 min. Helium was used as the carrier gas (flow rate: 4 mL min^{-1}). The mass spectrometry was operated in electron ionization (EI) mode (70 eV; interface temperature 380 °C; source temperature 340 °C). An emission current of 50 µA was used with the mass spectrometer acquiring in full scan mode (*m/z* 50 – 650 Daltons) at 0.8 s scan. Data acquisition and processing were conducted using Xcalibur software (3.0.63, Thermo Fisher Scientific). Peaks were identified using a combination of mass spectral information, GC retention times, and comparison to published datasets and the NIST mass spectral library (version 2.0).

GC-C-IRMS

A Thermo Finnigan Delta^{Plus} XP isotope ratio mass spectrometer was used to determine $\delta^{13}C$ values of FAME derivatized total lipid extracts. The mass spectrometer (EI, 100 eV, three Faraday cup collectors for *m/z* 44, 45 and 46) was interfaced to a Trace 2000 gas chromatograph via a Combustion III interface (CuO/NiO/Pt oxidation reactor maintained at 980 °C; Thermo Scientific). Samples were introduced *via* a PTV injector in spitless mode held at 200 °C onto a VF-23ms fused silica capillary column (60 m x 0.32 mm x 0.25 µm film thickness; Agilent Technologies), using helium carrier gas at 2.0 mL min^{-1}. The temperature programme comprised of an isothermal period of 2 min at 50°C increasing to 250°C at a rate of 10°C min^{-1}, followed by an isothermal period of 15 min at 250°C.

A standard FAME mixture (*n*-C_{11}, *n*-C_{13}, *n*-C_{16}, *n*-C_{21} & *n*-C_{23}) of known isotopic composition was used to assess instrument accuracy; samples were run in triplicate and the mean average $\delta^{13}C$ values of the ratio of $^{13}C/^{12}C$ were expressed relative to the Vienna Pee Dee Belemnite (VPDB). CO_2 of known isotopic composition was used as a reference gas to calibrate values. Data acquisition and processing were conducted using Isodat 3.0.94.12.

MALDI-TOF

Samples spotted in triplicate were analysed using a calibrated UltrafleXtreme III (Bruker Daltonics), with smartbeam-II laser, MALDI-ToF instrument in reflector mode. Spectral analysis was performed using the open-source software mMass[(3)] to identify individual peptides (Table S3, S4).

Peptide mass fingerprinting

MALDI-TOF data was visualised and processed in mMass (version 5.50). Baseline corrections and smoothing were applied with the default settings. Peaks were picked using a signal to noise ratio of 3.0–5.0 and then manually inspected.

Sacrificial Samples

Table S3: Peptide markers identified using ZooMS MALDI-TOF-MS following triboelectric sampling of Beast2Craft parchments.

Sample	Diagnostic markers present/ *m/z*	Species ID	References
**1765**	3034, 2883, 1180	Likely sheep	
**1786a**	2883, 1196, 1180	Sheep/goat	
**1786b**	2283, 1196, 1180	Sheep/goat	
**1786c**	2823	Sheep/goat	
**1790**	3034, 3017, 2883, 1196, 1180	Sheep	(4-9)
**1810**	3034, 3017, 2883, 1196, 1180	Sheep	
**1816**	3034, 3017, 2883, 1196, 1180	Sheep	
**1817**	3034, 2883, 1196, 1180	Sheep	
**1825**	3034, 3017, 2883, 1196, 1180	Sheep	

Chancery Rolls – UK Acts of Parliament

Table S4: Diagnostic peptide markers identified using ZooMS MALDI-TOF-MS following triboelectric sampling of Chancery Rolls documenting UK Acts of Parliament.

Sample	Diagnostic markers present/ *m/z*	Species ID	References
1814-1	3034, 3017, 2883, 1196, 1180	Sheep	
1814-2	3034, 3017, 2883, 1196, 1180	Sheep	
1814-3	3034, 3017, 2883, 1196, 1180	Sheep	
1814-4	3034, 3017, 2883, 1196, 1180	Sheep	
1814-5	3034, 3017, 2883, 1196, 1180	Sheep	
1815-1	3034, 3017, 2883, 1196, 1180	Sheep	
1815-2	3034, 3017, 2883, 1196, 1180	Sheep	
1815-3	3034, 3017, 2883, 1196, 1180	Sheep	
1815-4	3034, 3017, 2883, 1196, 1180	Sheep	
1815-5	3034, 3017, 2883, 1196, 1180	Sheep	
1815-6	3034, 3017, 2883, 1196, 1180	Sheep	
1816-1	3034, 3017, 2883, 1196, 1180	Sheep	
1816-2	3034, 3017, 2883, 1196, 1180	Sheep	
1816-3	3034, 3017, 2883, 1196, 1180	Sheep	
1816-4	3034, 3017, 2883, 1196, 1180	Sheep	
1816-5	3034, 3017, 2883, 1196, 1180	Sheep	
1817-1	3034, 3017, 2883, 1196, 1180	Sheep	
1817-2	3034, 2883, 1196, 1180	Sheep	(4–9)
1817-3	3034, 2883, 1196, 1180	Sheep	
1817-4	3034, 3017, 2883, 1196, 1180	Sheep	
1817-5	3034, 3017, 2883, 1196, 1180	Sheep	
1818-1	3034, 2883, 1196, 1180	Sheep	
1818-2	3034, 2883, 1196, 1180	Sheep	
1818-3	3034, 3017, 2883, 1196, 1180	Sheep	
1818-4	3034, 3017, 2883, 1196, 1180	Sheep	
1818-5	3034, 3017, 2883, 1196, 1180	Sheep	
1819-1	3034, 3017, 2883, 1196, 1180	Sheep	
1819-2	3034, 3017, 2883, 1196, 1180	Sheep	
1819-3	3034, 3017, 2883, 1196, 1180	Sheep	
1819-4	3034, 3017, 2883, 1196, 1180	Sheep	
1819-5	3034, 3017, 2883, 1196, 1180	Sheep	
1820-1	3034, 3017, 2883, 1196, 1180	Sheep	
1820-2	3034, 3017, 2883, 1196, 1180	Sheep	
1820-3	3034, 3017, 2883, 1196, 1180	Sheep	
1820-4	3034, 3017, 2883, 1196, 1180	Sheep	
1820-5	3034, 3017, 2883, 1196, 1180	Sheep	

Statistical analysis

Statistical treatment of the data generated during this study was carried out using SPSS 29.0.1.0 software. The Mann-Whitney U Test was used (Tables S5 – S7) to compare the area densities (µg cm^{-2}) of differing extractions and efficacy of invasive and vacuum-aided extractions.

Table S5: Mann-Whitney U test for the two-tailed comparison of the vacuum-aided horizontal edge (Region 1, extraction D) and invasive horizontal edge (Region 1, INV) area densities (µg cm^{-2}). Null hypothesis: no significant difference between the two groups at p < .05.

**Ranks**	**Vacuum Horizontal Edge**	**Invasive Horizontal Edge**
Observations	9	9
Mean Rank	6.6	12.4
Sum of Ranks	59	112
**Statistic**	**Vacuum Horizontal Edge vs. Invasive Horizontal Edge**	
Mann-Whitney U	**14.0**	
Z	**-2.3**	
p-value	**.02**	

Table S6: Mann-Whitney U test for the two-tailed comparison of the vacuum-aided centre (Region 3, extraction E) and vacuum-aided horizontal edge (Region 1, extraction D) area densities (µg cm^{-2}). Null hypothesis: no significant difference between the two groups at p < .05.

**Ranks**	**Vacuum Centre**	**Vacuum Horizontal Edge**
Observations	9	9
Mean Rank	12.1	6.9
Sum of Ranks	109	62
**Statistic**	**Vacuum Centre vs. Vacuum Horizontal Edge**	
Mann-Whitney U	**62**	
Z	**-2.1**	
p-value	**.04**	

Table S7: Mann-Whitney U test for the two-tailed comparison of the vacuum-aided centre (Region 3, extraction E) and vacuum-aided vertical margin (Region 2, extraction A, B, C) area densities (µg cm-$^{2)}$. Null hypothesis: no significant difference between the two groups at p < .05.

**Ranks**	**Vacuum Centre**	**Vacuum Vertical Margin (A)**
Observations	9	27
Mean Rank	22.6	16.4
Sum of Ranks	203	427
**Statistic**	**Vacuum Centre vs. Vacuum Vertical Margin (A)**	
Mann-Whitney U	**76**	
Z	**-1.5**	
p-value	**.13**	

Lipid heterogeneity within the same parchment

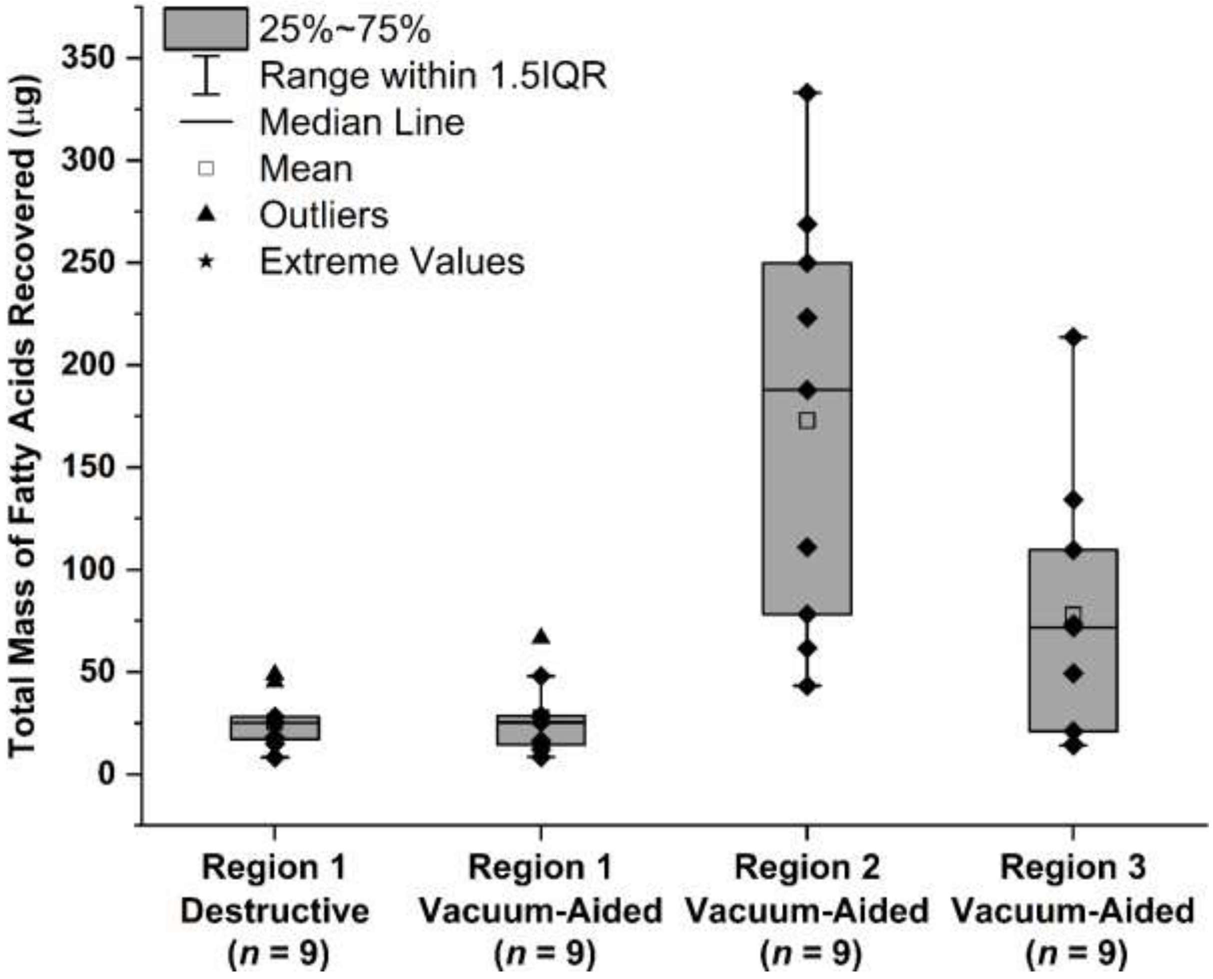

Figure S2: Box-and-whisker plot comparing the total mass of fatty acid recovery (µg) from parchments dated 1765 – 1825 CE. The 'edge' region (Region 1) comprised a single vacuum-aided and invasive extraction (1 cm^2 and 3.14 cm^2, respectively); the 'vertical margin' region (Region 2, 3.14 cm^2) comprised three vacuum-aided extractions (A, B, C, 9.42 cm^2) which aliquots of were combined to give one total lipid extract. The 'centre' region (Region 3, 3.14 cm^2) comprised a single vacuum-aided extraction. The horizontal line within each box indicates the median value area density and the square data plot indicates the mean average value. The lower and upper boundaries of the boxes indicate the 25th and 75th percentiles, respectively. Data points are shown as diamonds while outliers and extreme outliers are identified as triangular and asterisk data plots greater than 1.5x and 3x the interquartile range, respectively. Whiskers above and below the boxes extend to the largest and smallest values within 1.5x the interquartile range.

References

(1) Collins, M. J. “B2C Parchment Collection,” can be found under https://sites.google.com/palaeome.org/ercb2c/get-involved/b2c-parchment-collection, **2023** (accessed 1 February 2024).

(2) The National Archives. "The National Archives Website: Discovery: C 65, Chancery: Parliament Rolls 1327-2010,” can be found under https://discovery.nationalarchives.gov.uk/details/r/, **2023** (accessed 1 February 2024).

(3) Strohalm, M.; Kavan, D.; Novák, P.; Volný, M.; Havlíček, V. mMass 3: A Cross-Platform Software Environment for Precise Analysis of Mass Spectrometric Data. *Anal. Chem.* **2010**, *82* (11), 4648–4651. https://doi.org/10.1021/ac100818g.

(4) Buckley, M.; Collins, M.; Thomas-Oaies, J.; Wilson, J. C. Species Identification by Analysis of Bone Collagen Using Matrix-Assisted Laser Desorption/Ionisation Time-of-Flight Mass Spectrometry. *Rapid Commun. Mass Spectrom.* **2009**, *23* (23), 3843–3854. https://doi.org/10.1002/rcm.4316.

(5) Buckley, M.; Whitcher Kansa, S.; Howard, S.; Campbell, S.; Thomas-Oates, J.; Collins, M. Distinguishing between Archaeological Sheep and Goat Bones Using a Single Collagen Peptide. *J. Archaeol. Sci.* **2010**, *37* (1), 13–20. https://doi.org/10.1016/j.jas.2009.08.020.

(6) Buckley, M.; Collins, M.; Thomas-Oates, J.; Wilson, J. C. Erratum: Species Identification by Analysis of Bone Collagen Using Matrix-Assisted Laser Desorption/Ionisation Time-of-Flight Mass Spectrometry. *Rapid Commun. Mass Spectrom.* **2010**, *24* (22), 3372–3372. https://doi.org/10.1002/rcm.4745.

(7) Kirby, D. P.; Buckley, M.; Promise, E.; Trauger, S. A.; Holdcraft, T. R. Identification of Collagen-Based Materials in Cultural Heritage. *Analyst* **2013**, *138* (17), 4849–4858. https://doi.org/10.1039/C3AN00925D.

(8) Welker, F.; Hajdinjak, M.; Talamo, S.; Jaouen, K.; Dannemann, M.; David, F.; Julien, M.; Meyer, M.; Kelso, J.; Barnes, I.; Brace, S.; Kamminga, P.; Fischer, R.; Kessler, B. M.; Stewart, J. R.; Pääbo, S.; Collins, M. J.; Hublin, J. J. Palaeoproteomic Evidence Identifies Archaic Hominins Associated with the Châtelperronian at the Grotte Du Renne. *Proc. Natl. Acad. Sci. USA.* **2016**, *113* (40), 11162–11167. https://doi.org/10.1073/pnas.1605834113.

(9) Buckley, M.; Harvey, V. L.; Chamberlain, A. T. Species Identification and Decay Assessment of Late Pleistocene Fragmentary Vertebrate Remains from Pin Hole Cave (Creswell Crags, UK) Using Collagen Fingerprinting. *Boreas* **2017**, *46* (3), 402–411. https://doi.org/10.1111/bor.12225.
